# Supplementary material for: Methylation-regulated decommissioning of multimeric PP2A complexes
Source: Nat Commun. 2017 Dec 22;8:2272. doi: 10.1038/s41467-017-02405-3 (PMC5741625; doi:10.1038/s41467-017-02405-3)
Supplement: Supplementary file 1 — Supplementary Information [file 41467_2017_2405_MOESM1_ESM.pdf]

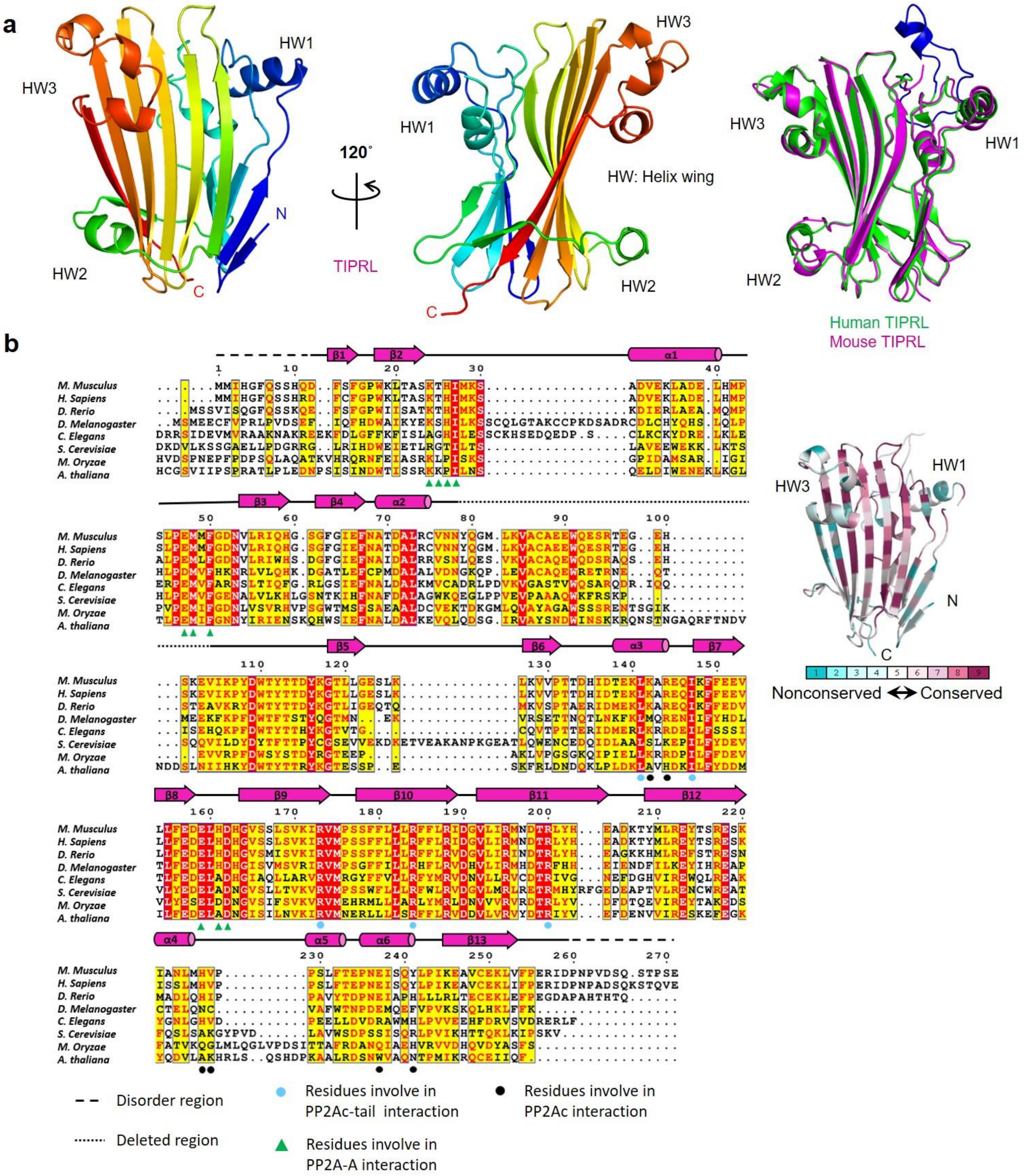

Supplementary Figure 1. Structural and sequence conservancy of TIPRL. (a) Overall structure of mouse TIPRL in two views differed by 120° in blue-to-red rainbow colors from N- to C-termini. The structural overlay with human TIPRL (PDB code: 5D9G) is shown (right). The missing structure resulted from internal deletion in Mouse TIPRL presented in Human TIPRL was colored blue. (b) Sequence alignment of TIPRL from diverse species from yeast to human, with

secondary structures indicated above and residues involved in interactions with PP2Ac and the scaffold A-subunit indicated below. Red and yellow boxes highlight identical and similar residues, respectively. The levels of residue conservation were calculated by ConSurf Server and color-coded on the structure of TIPRL from the core enzyme-bound complex (right).

a

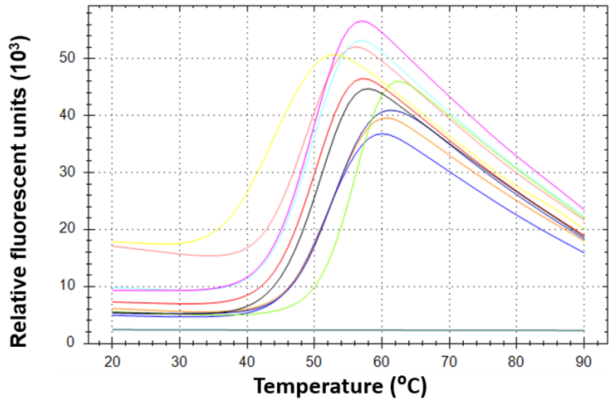

TIPRL	T_m(ºC)	Color
WT	51	
L141E	50	
K142D	50	
R144E	50	
R173E	53	
L182R	44.3 ± 0.3	
R184E	55	
R200E	49	
L231D	52.6 ± 0.7	
Y241D	52	

b

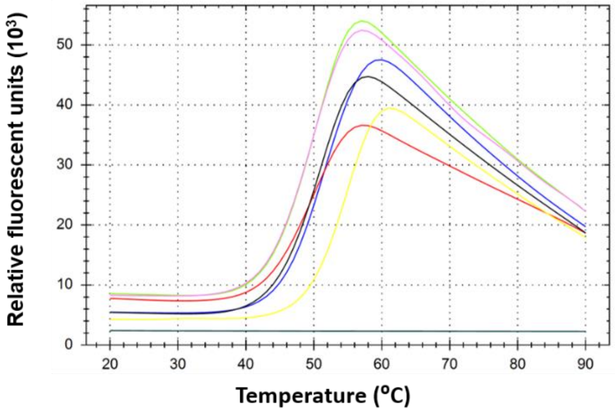

TIPRL	T_m(ºC)	Color
WT	51	
H26A	52.6 ± 0.7	
I27D	50	
M48R	50	
E159R	55.7 ± 0.3	
D162R	51	

Supplementary Figure 2. Thermal stability of WT and mutant TIPRL. The melting curves for TIPRL WT and mutants used in examination of the interactions between TIPRL and PP2A-A (a) or PP2Ac (b) subunit. All protein samples (1mg/ml) and negative control (no protein) were treated by an increasing gradient of temperature (1ºC per minute) from 20ºC to 90 ºC in the presence of olive green whose fluorescent signals would increase upon association with hydrophobic residues exposed during protein unfolding. The right table shows the color code for the melting curves of corresponding TIPRL mutants, and their unfolding transition temperature (T_m) presented as mean ± SEM. The majority of TIPRL mutants have similar T_m as WT except E159R and R184E that have a T_m ~4ºC higher, and L182R that has a T_m 6ºC less than WT. Although the thermal stability of L182R is reduced, the interaction between TIPRL and PP2Ac is not affected by this mutation (Fig. 3c).

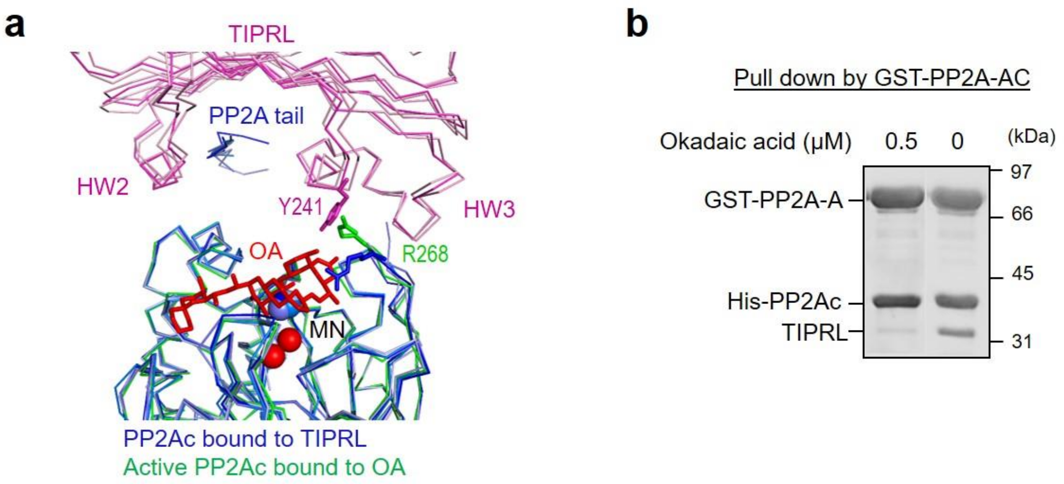

Supplementary Figure 3. Effects of okadaic acid (OA) to the interaction between TIPRL and PP2A core enzyme. (a) Overlay of crystal structures of active PP2Ac bound to OA (green, PDB code: 2IE4) and PP2Ac bound to TIPRL (blue). The close-up view of interaction interface is shown. Manganese ions in active and TIPRL-bound PP2Ac are shown in red and blue spheres, respectively. Structure of OA is shown in red stick. (b) Pulldown of TIPRL by GST-PP2A core enzyme in the presence or absence of okadaic acid.

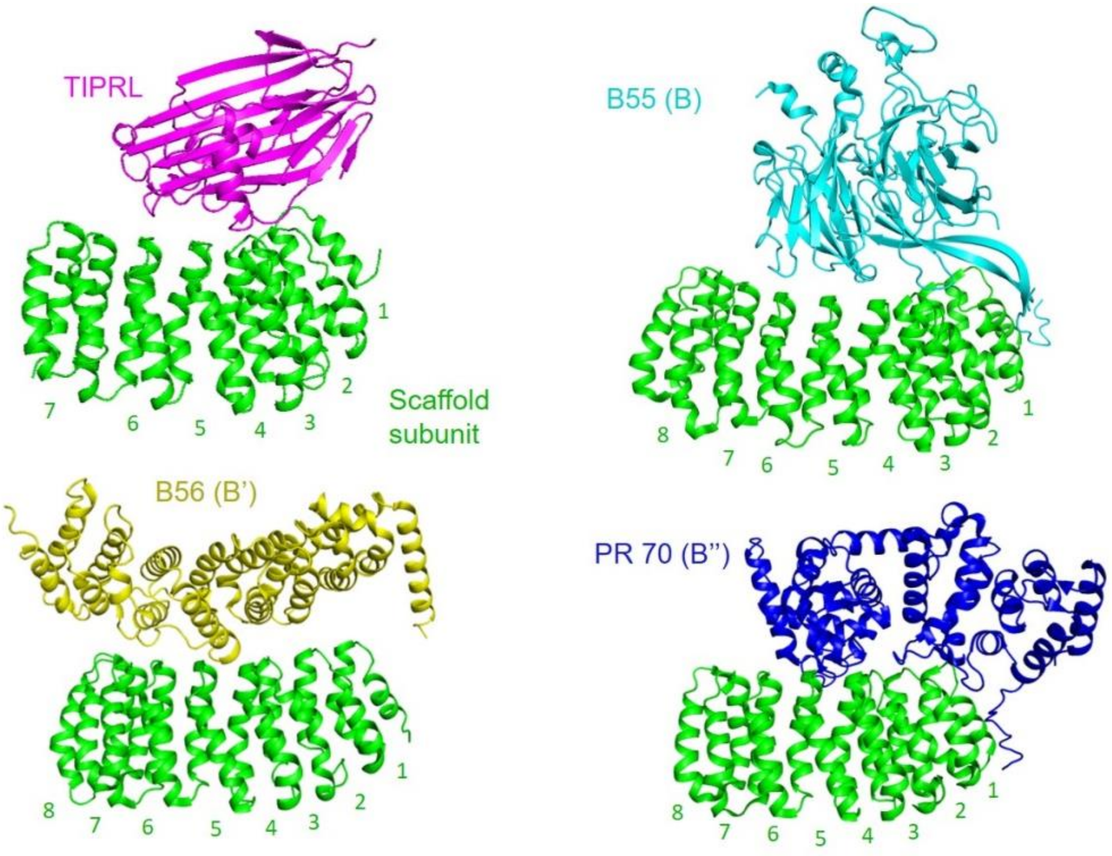

Supplementary Figure 4. TIPRL binding to the scaffold subunit overlaps with regulatory subunits. Cartoon representation of interaction between TIPRL (magenta), B/B55 (cyan), B'/B56 (yellow), B"/PR70 (blue) and the scaffold A-subunit. The N-terminal HEAT repeats 1-8 of the scaffold subunit were indicated by numbers.

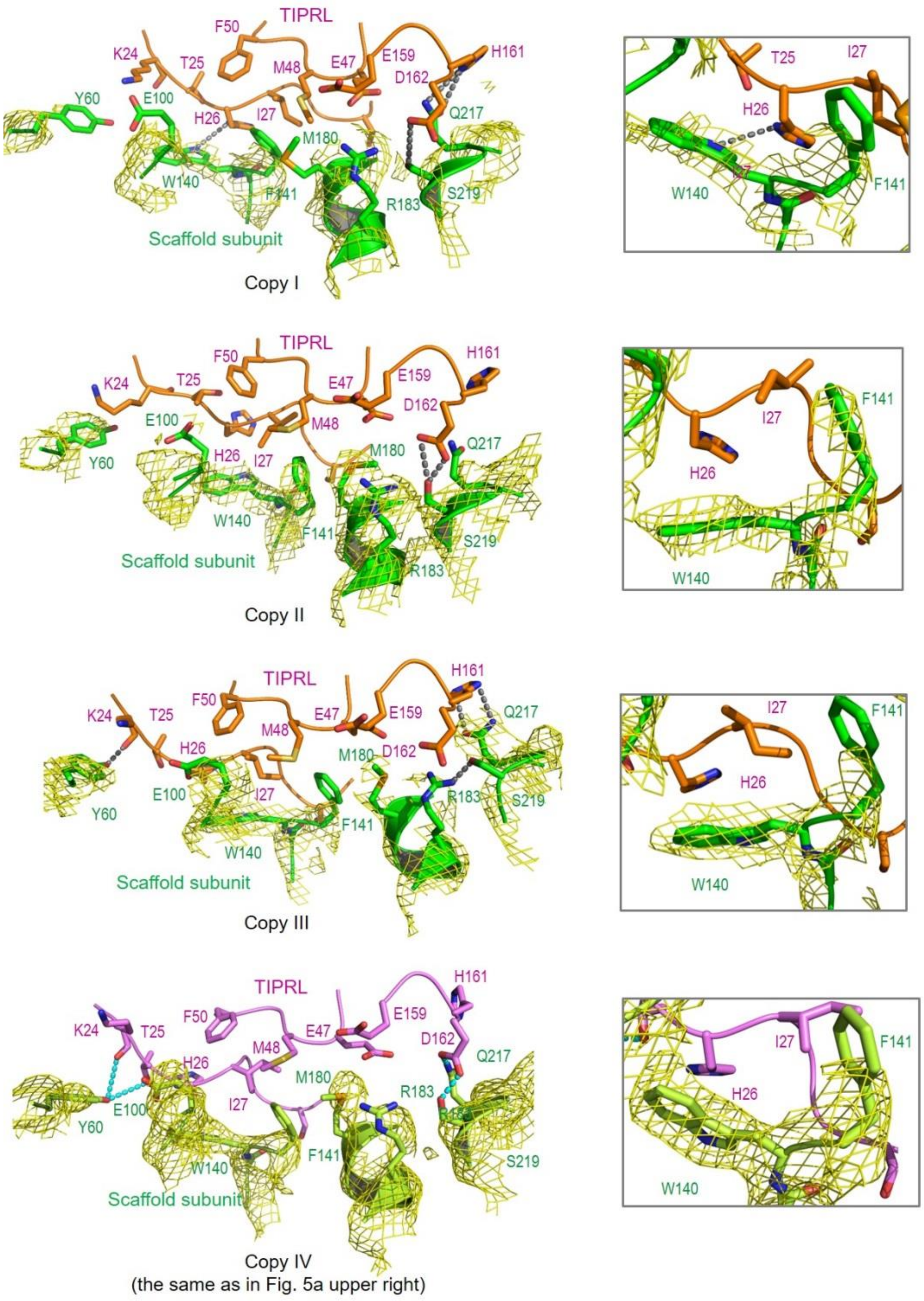

Supplementary Figure 5. Side-by-side views of interactions between TIPRL and the scaffold subunit in different copies of the PP2A-TIPRL complex in the asymmetric unit. Close-up views of interactions between TIPRL and the scaffold A-subunit in each of the four different copies in the asymmetric unit (Copy I, II, III, IV). Copy IV is the same as shown in Fig. 5a upper right panel. The F_O-F_C omit maps for the scaffold subunit residues at the interface with TIPRL is contoured in yellow mesh at 1.5 σ for Copy I, II, III, and 2.0 σ for Copy IV. Inlets are zoom-in views to highlight the omit map at 2.0 σ for sidechains of the scaffold subunit.

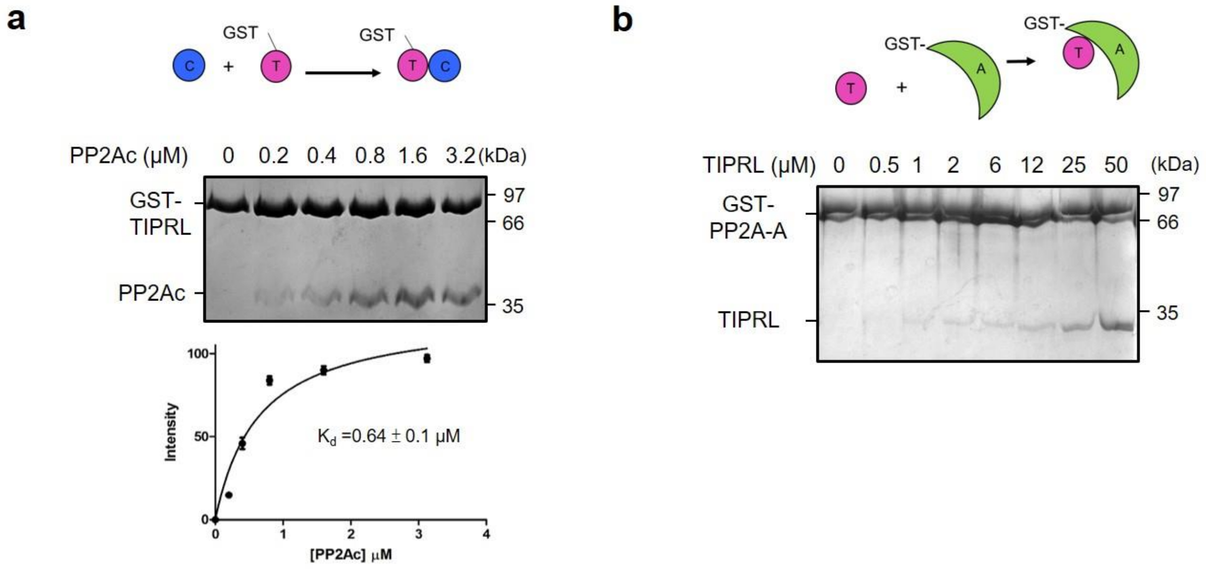

Supplementary Figure 6. Binding affinities of TIPRL to PP2A scaffold subunit and PP2Ac. (a) Pulldown of GST-TIPRL with titrated concentrations of PP2Ac. The bound proteins were examined on SDS-PAGE. The intensity of bound PP2Ac was estimated using image J. Results from three independent experiments were used to fit the intensity of bound PP2Ac (Y-axis) versus PP2Ac concentrations into one site specific binding model in Prism (GraphPad Prism). The K_d is calculated to be 0.64 μM. (b) Pulldown of GST-PP2A scaffold subunit with titrated concentrations of TIPRL. The bound proteins were examined on SDS-PAGE. The K_d is estimated to be approximately 10 μM.

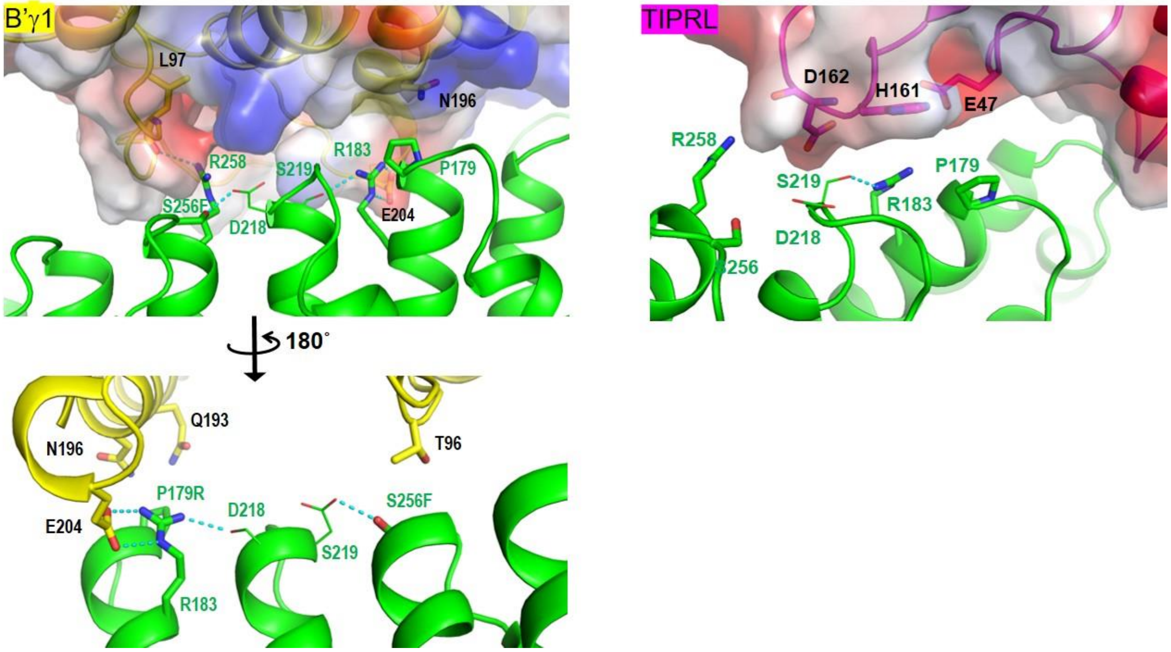

Supplementary Figure 7. Illustration of disease mutations at the shared interfaces of the B' regulatory subunit and TIPRL to the PP2A scaffold subunit. Close-up views of interaction interface between B'γ1 and the scaffold subunit in the structure of PP2A-B'γ1 holoenzyme (upper left), and between TIPRL and the scaffold subunit of the structure of PP2A-TIPRL (upper right). Cancer and intellectual disable mutations on the scaffold subunit were drawn in sticks and inter-intra molecular interactions were labeled with dashed lines. The contours of the surface of B'γ1 and TIPRL were shown. The blue and red regions of the surface indicate the nitrogen and oxygen exposed to the surface of the structure. The lower panel is the close-up opposite view of the interaction mode between B'γ1 and the scaffold A-subunit.

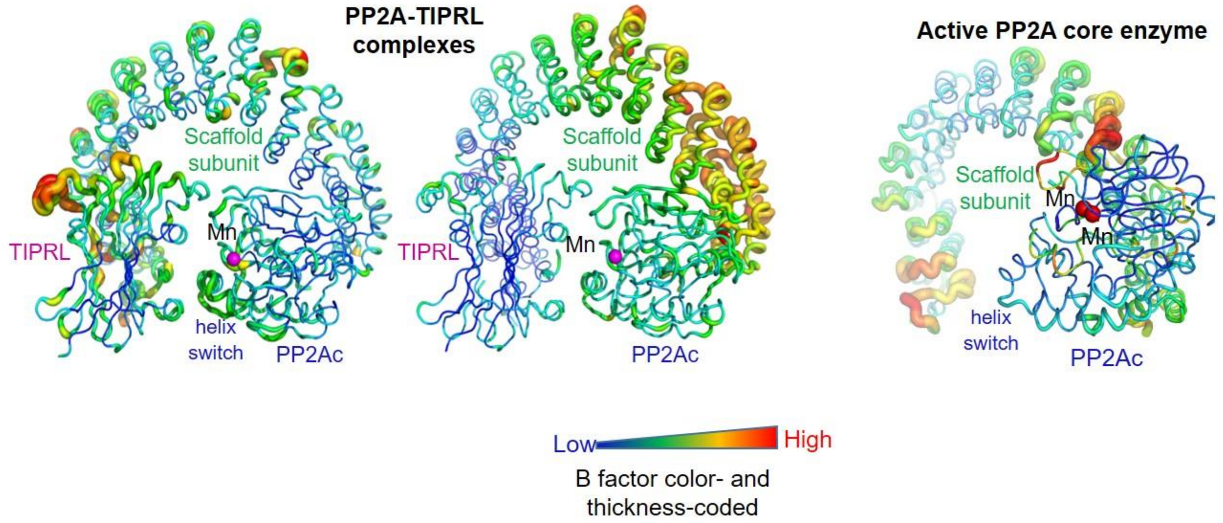

Supplementary Figure 8. B factors of the PP2A-TIPRL complexes in the crystallographic asymmetric unit compared to the active PP2A core enzyme. The level of the thickness and distinct colors reflect the values of the B factors of the local structure. Representative distribution patterns of the B factors of heterotrimers were shown. The left panel shows a heterotrimer that has a stable global structure but dynamic local structures at helix switch of PP2Ac and at the interface between PP2A scaffold subunit and TIPRL. The structure in the middle panel, on the other hand, displays more stable structure of TIPRL and helix switch of PP2Ac, but has more dynamic global structure especially at the A-PP2Ac interface. These indicate that the binding of TIPRL with core enzyme may cause structural fluctuations and destabilize their local and global structures. The right panel shows the distribution of B factors of the structure of active PP2A core enzyme. The overall B factors are low through PP2Ac including the helix switch.

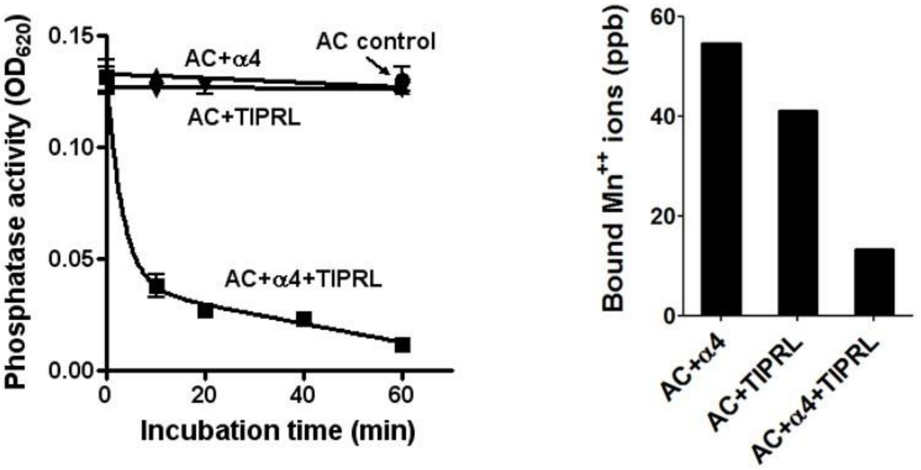

Supplementary Figure 9. Time-dependent changes of the phosphatase activity of the PP2A core enzyme stabilized by the presence of excess amount of free Mn^{2+} during co-incubation with α4 alone, TIRPL alone, or α4/TIPRL together. All experiments were performed in triplicate and repeated three times. Mean ± s.e.m. were calculated (left panel). At the end of incubation, the levels of protein-bound Mn^{2+} were examined by ICP-MS after removal of free Mn^{2+} by gel filtration chromatography (right).

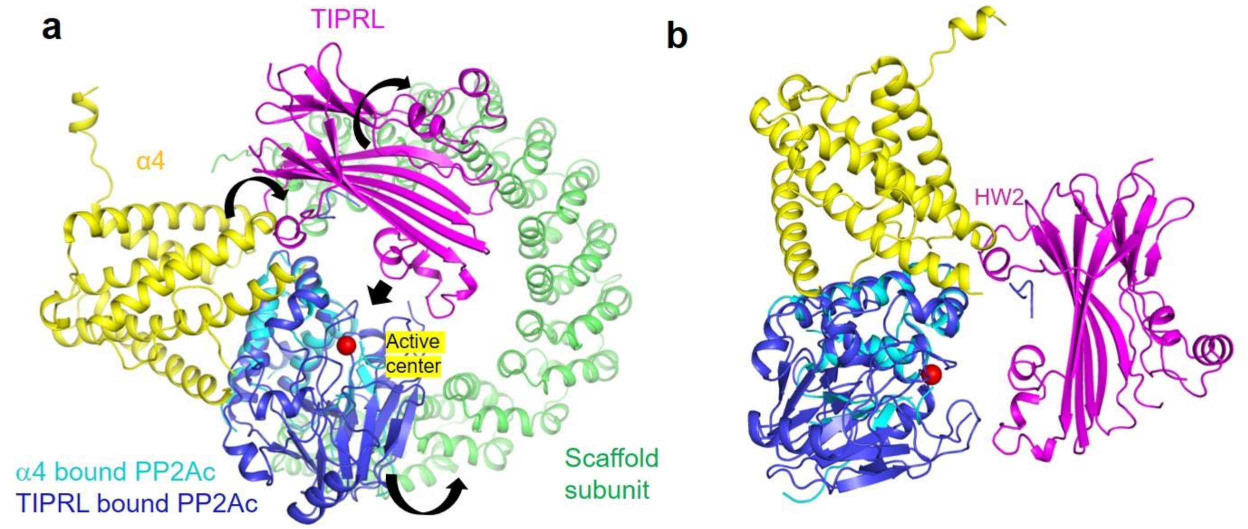

Supplementary Figure 10. Predicted structure of the recycling complex (PP2Ac-α4-TIPRL). (a) Structural overlay of the PP2A-TIPRL complex with partial PP2Ac bound to α4 aligned by the N-terminal helix motif of PP2Ac. Black arrows indicate potential forces in the complex prior to global changes of PP2Ac. The scaffold subunit would be expelled by global allosteric changes in PP2Ac. (b) The structural model of the PP2Ac-α4-TIPRL complex built based on structural overlay in (a). Notably, there are steric clashes between HW2 of TIPRL and α4. Conformational changes in α4 and TIPRL are expected to occur during holoenzyme disassembly and formation of the PP2Ac-α4-TIPRL complex.

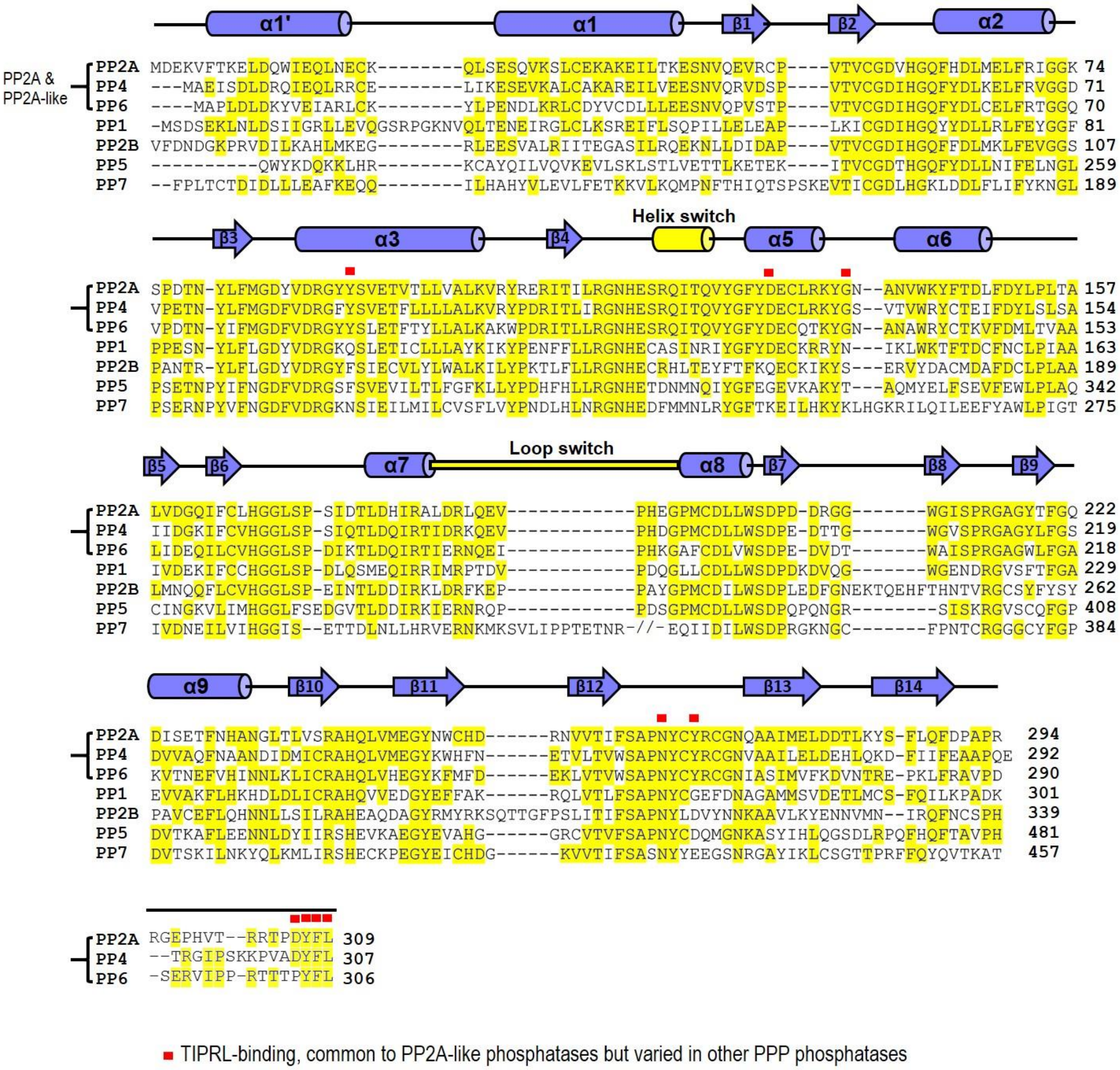

Supplementary Figure 11. Sequence alignment of the catalytic subunit of PP2A with other members of the PPP family Ser/Thr phosphatases of human origin. Secondary structural elements of the catalytic subunit of PP2A are indicated above the sequences. Conserved residues are highlighted in yellow. Residues that participate in TIPRL-binding are indicated by red squares above the sequence. TIPRL-binding residues in PP2A are common or highly similar to PP2A-like phosphatases but varied in other members of the PPP family phosphatases.

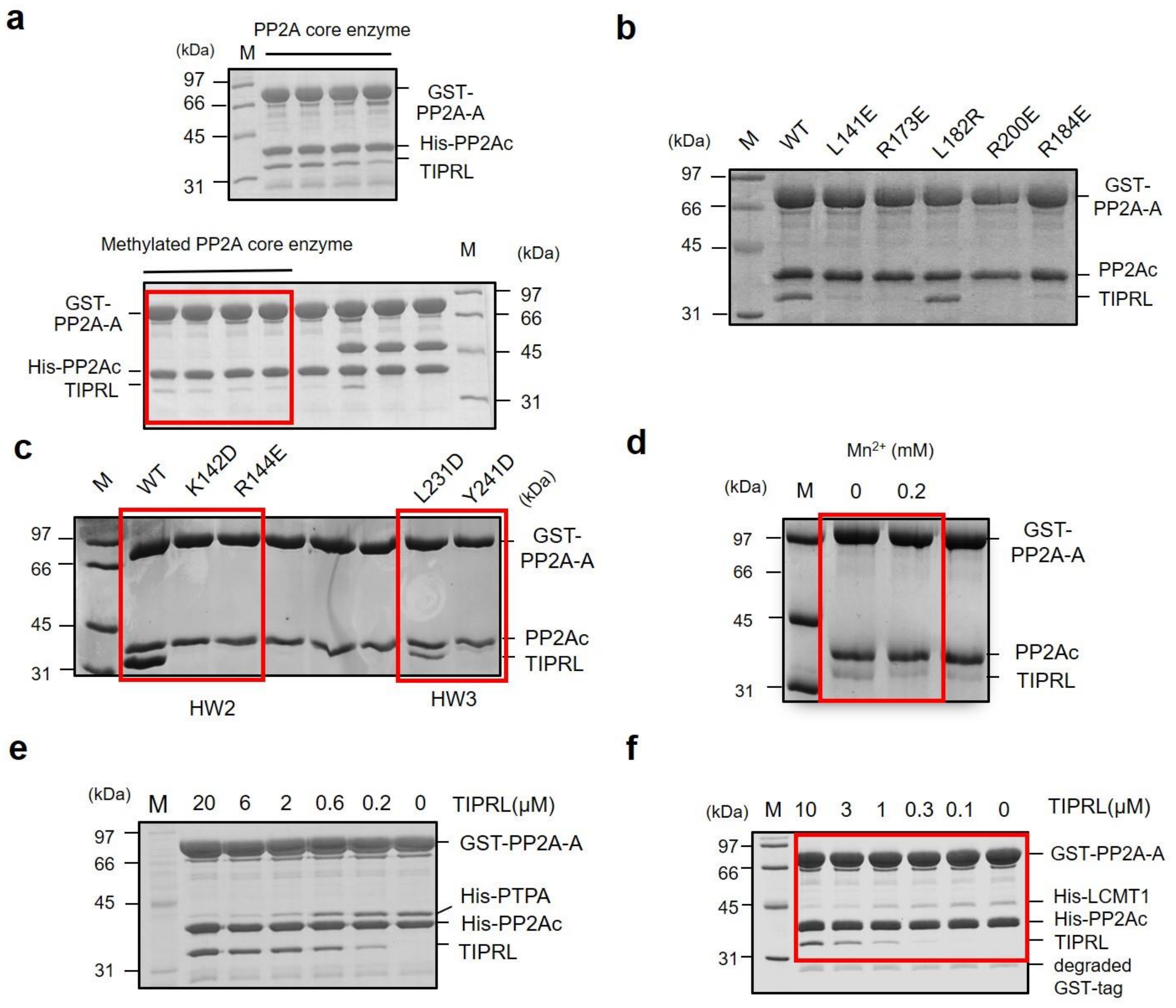

Supplementary Figure 12. Uncropped images of SDS-PAGE shown in figures (a) 2f, (b) 3c, (c) 3d, (d) 3e, (e) 4b, and (f) 4c. Red boxes highlight the portions of the gels that are shown in the figures. “M” stands for “marker”.

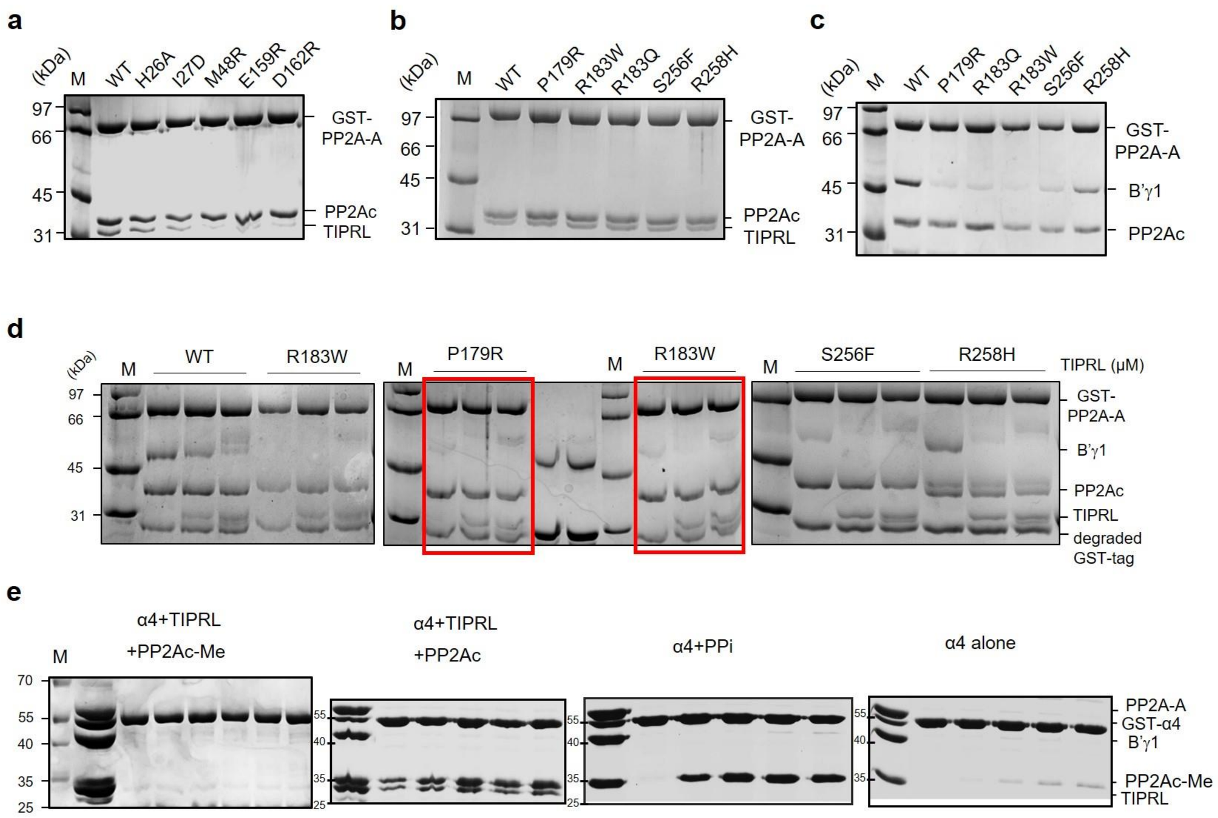

Supplementary Figure 13. Uncropped images of the SDS-PAGEs shown in figures (a) 5c, (b) 5e, (c) 5f, (d) 5g, and (e) 6b. Red boxes highlight the portions of the middle gel in **(d)** that are shown in the figures. "M" stands for "marker".

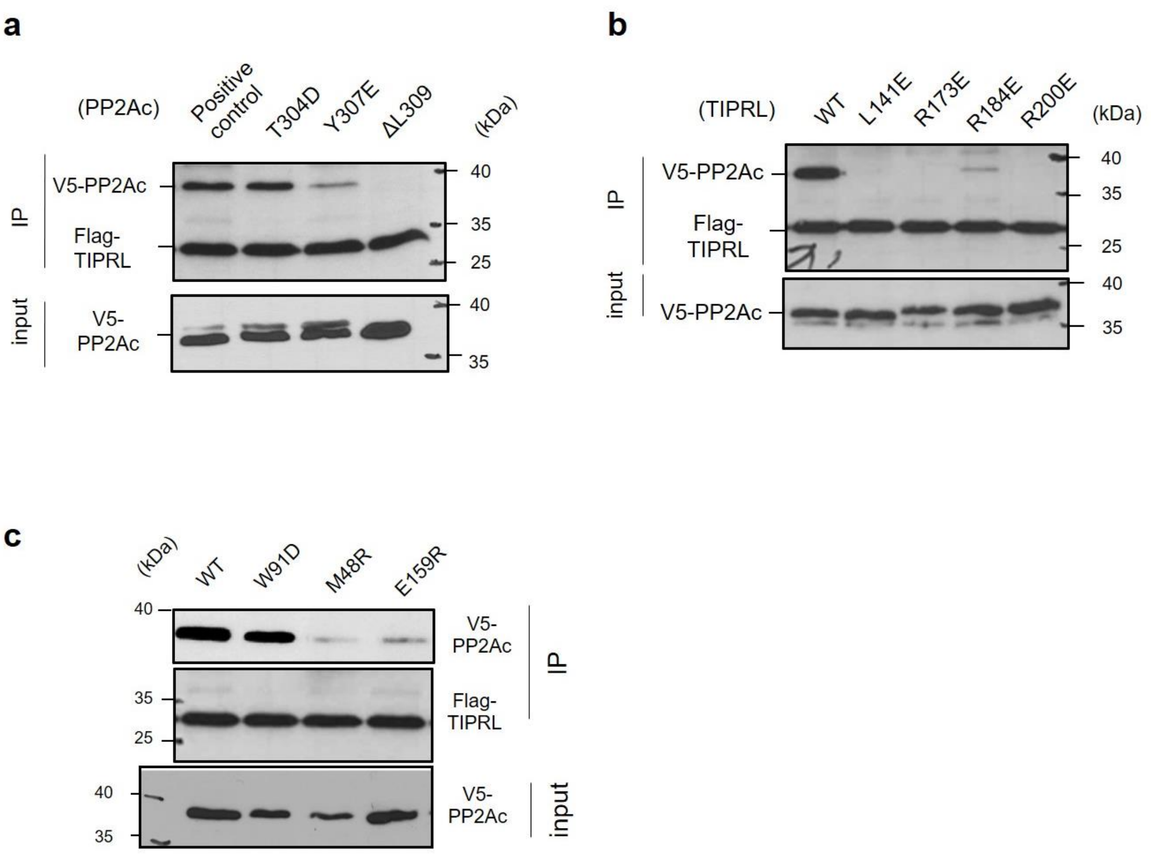

Supplementary Figure 14. Images of western blot shown in figure (a) 3a, (b) 3b, and (c) 5c with markers.

Supplementary Table 1 Oligonucleotide primers used in this study.

**Name**	Forward Sequence (5'->3')<br>Reverse Sequence (5'->3')
TIPRL_12-259 (delete 1-11 and 260-271)	cgagaatctttattttcagggatccgacttctccttcgggcc
	aagaaagctgggtcctaggcggccgcttattctgagggcgtactttgtgag
TIPRL _delete (94-103)	gcttgtgctgaagagtggcaggaagttattaaaccatatgactggacctatacaacag
	ctgttgtataggtccagtcatatggtttaataacttcctgccactcttcagcacaagc
TIPRL _H26A	ctgacggcgtccaagaccgcgatcatgaagtctgcggatgtgg
	ccacatccgcagacttcatgatcgcggtcttggacgccgtcag
TIPRL _I27D	cggcgtccaagacccacgatatgaagtctgcggatgtggaaaagttagc
	gctaacttttccacatccgcagacttcatatcgtgggtcttggacgccg
TIPRL _M48R	atgccatccctccctgaaaggatgtttggagacaa
	ttgtctccaaacatcctttcagggagggatggcat
TIPRL _W91D	gtgctgaagaggatcaggaaagtaggacggaggg
	cgtcctactttcctgatcctcttcagcacaagctac
TIPRL _L141E	cctacaacagatcatatagatacagaaaaagagaaagccagagaacagattaaa
	tttaatctgttctctggctttctctttttctgtatctatatgatctgttgtagg
TIPRL _K142D	caacagatcatatagatacagaaaaattggatgccagagaacagattaagtttttgaag;
	cttcaaaaaacttaatctgttctctggcatccaatttttctgtatctatatgatctgttg
TIPRL _R144E	cagatcatatagatacagaaaaattgaaagccgaagaacagattaagtttttgaagaag
	cttcttcaaaaaacttaatctgttcttcggctttcaatttttctgtatctatatgatctg
TIPRL _E159R	ctgtttgaagatagattgcatgatcatggtgtttccag
	caccatgatcatgcaatctatcttcaaacaggagaacttcttc
TIPRL _D162R	gaagatgaattgcatcgtcatggtgtttccag
	gaaacaccatgacgatgcaattcatcttcaaacaggagaac
TIPRL_R173E	tggtgtttccagcctgagtgtgaaaattgaggtgatgccttccagc
	gctggaaggcatcacctcaattttcacactcaggctggaaacacca
TIPRL_L182R	ttccagcttctttctgcggttgcggtttttcctga
	tcaggaaaaaccgcaaccgcagaaagaagctggaa
TIPRL_R184E	ccagcttctttctgctgttggagtttttcctgagaattgatg
	aattctcaggaaaaactccaacagcagaaagaagctgg
TIPRL_R200E	catcagaatgaatgacacggagctttaccatgaggctgac
	gtcagcctcatggtaaagctccgtgtcattcattctgatg
TIPRL_L231D	gctaatttaatgcatgttccaccttccgatttcacggaacctaatgaaatatcac
	gtgatatttcattaggttccgtgaaatcggaaggtggaacatgcattaaattagc
TIPRL_Y241D	cttcacggaacctaatgaaatatcacaagacttaccaattaaggaagcagtttgtgag
	ctcacaaactgcttccttaattggtaagtcttgtgatatttcattaggttccgtgaag
PP2Aa_delete (1-7)	cgagaatctttattttcagggatccatggcggcggcc
	aagaaagctgggtcctaggcggccgctcaggcgagagacagaacagtc
PP2Aa_P179R	cggaacctgtgctcagatgacacccgaatggtgcggcgggccg

	cggcccgccgcaccattcgggtgtcatctgagcacaggttccg
PP2Aa_R183W	cacccccatggtgcggtgggccgcagcctccaagc
	gcttggaggctgcggcccaccgcaccatgggggtg
PP2Aa_R183Q	cacccccatggtgcggcaagccgcagcctccaagc
	gcttggaggctgcggcttgccgcaccatgggggtg
PP2Aa_S256F	gccaggccgctgaagacaagttctggcgcgtccgctacatgg
	ccatgtagcggacgcgccagaacttgtcttcagcggcctggc
PP2Aa_R258H	ggccgctgaagacaagtcctggcacgtccgctacatggtggctgacaag
	cttgtcagccaccatgtagcggacgtgccaggacttgtcttcagcggcc
PP2Ac_T304D	gtagaggcgaactcgtcgtgacccagactacttcctttaaggatcc
	ggatccttaaaggaagtagtctgggtcacgacgagttcgcctctac
PP2Ac_Y307E	ctcgtcgt accccagacgagttcctttaaggatccggaattccg
	cggaattccggatccttaaaggaactcgtctggggtacgacgag
PP2Ac_delete 309	cgtcgtaccccagactacttctaaggatccggaattccgcc
	ggcggaattccggatccttagaagtagtctggggtacgacg
