# Supplementary material for: Methylation-regulated decommissioning of multimeric PP2A complexes
Source: Nat Commun. 2017 Dec 22;8:2272. doi: 10.1038/s41467-017-02405-3 (PMC5741625; doi:10.1038/s41467-017-02405-3)
Supplement: Supplementary file 3 — Description of Additional Supplementary Files [file 41467_2017_2405_MOESM3_ESM.pdf]

Description of Supplementary Files

File Name: Supplementary Movie 1
Description: **Morph of diverse conformations of PP2A scaffold subunit in different multimeric complexes.** Three representative PP2A holoenzymes from B (PDB code: 3DW8), B' (PDB code: 2NPP), and B" (PDB code: 4I5L) families and the PP2A core enzyme (PDB code: 2IE4) were used to morph the trajectories of scaffold subunit conformational changes among different multimeric complexes.
